# Recent advances in immunotherapy for lung cancer

**DOI:** 10.1002/cai2.55

**Published:** 2023-02-24

**Authors:** Qi Wang, Chunxia Su, Caicun Zhou

**Affiliations:** ^1^ Department of Oncology, Shanghai Pulmonary Hospital Tongji University Shanghai China

**Keywords:** nonsmall cell lung cancer, small cell lung cancer, perioperative period, late stage, new immunotherapy

## Abstract

Lung cancer is the malignant tumor with the highest morbidity and mortality in China, and nonsmall cell lung cancer is a common form of lung cancer. After undergoing chemotherapy and molecular targeted therapy, the treatment of lung cancer has now fully entered the era of immunotherapy. Immunotherapy‐based treatment has become one of the standard treatments for lung cancer. Immunotherapy has also gradually moved from the back line to the front line, from advanced to early patients. This article focuses on the latest developments in perioperative and advanced lung cancer immunotherapy, discusses the problems and challenges at the current stage, and explores new directions for future development.

## Graphical abstract

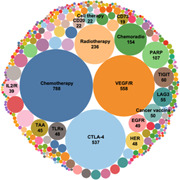

**Abbreviations**
ADC: antibody drug conjugate
BSC: best supportive care
DCR: disease control rate
DFS: disease‐free survival
DLL3: delta‐like ligand 3
ESMO: European Society of Medical Oncology
HLE BiTE®: half‐life‐extended bispecific T‐cell engager
ICI: immune checkpoint inhibitor
NSCLC: nonsmall cell lung cancer
ORR: objective response rate
PARP: poly ADP‐ribose polymerase
pCR: pathological complete remission
PD‐1: programmed death protein‐1
PD‐L1: programmed death ligand‐1
PFS: progression‐free survival
WCLC: World Conference on Lung Cancer

## 1 INTRODUCTION

Lung cancer is a tumor with the highest morbidity and mortality rate in the world. Nonsmall cell lung cancer (NSCLC) is an important pathological type of lung cancer, accounting for about 85% of lung cancer [1, 2]. After undergoing chemotherapy and molecular targeted therapy, the treatment of lung cancer has now fully entered the era of immunotherapy [3]. Immune checkpoint inhibitors (ICIs) targeting programmed death protein‐1 (PD‐1) or programmed death ligand‐1 (PD‐L1), have brought great changes to the treatment of NSCLC in the past 10 years. In the field of lung cancer, a variety of PD‐1/PD‐L1 monoclonal antibodies have been approved for clinical use. In recent years, immunotherapy has gradually moved from the back line to the front line, from advanced to early patients. This article focuses on the latest developments in perioperative and advanced lung cancer immunotherapy, discusses the problems and challenges at the current stage, and explores new directions for future development.

## 2 ADVANCES IN IMMUNOTHERAPY FOR NSCLC

### 2.1 Advances in perioperative immunotherapy

In recent years, the treatment pattern of NSCLC patients has undergone tremendous changes. Immunotherapy has gradually moved forward from the advanced stage to the early stage, which is expected to improve the cure rate of early‐stage NSCLC patients. Several clinical trials are currently being carried out, including immune neoadjuvant therapy and postoperative immune adjuvant therapy. Many clinical trials are still in progress, and the results of these studies will determine the future treatment model. We expect that these research data will contribute to the perioperative treatment of lung cancer.

#### 2.1.1 Adjuvant immunotherapy

The IMpower010 [4] study aimed to explore the effectiveness of ICI as adjuvant therapy for patients with early‐stage lung cancer who have completed radical treatment (including surgery and postoperative adjuvant chemotherapy). The results of the study published in “LANCET” in 2021 showed that atezolizumab adjuvant immunotherapy compared with Best Supportive Care (BSC), significantly improved the disease‐free survival (DFS) in patients with Stage II–IIIA NSCLC (hazard ratio [HR] = 0.79, 95% confidence interval [CI]: 0.64–0.96, *p* = 0.002). Subgroup analysis suggested that in PD‐L1 positive (TPS ≥ 1%) Stage II–IIIA patients, the efficacy reached a significant statistical difference (HR = 0.66, 95% CI: 0.50–0.88, *p* = 0.004). The updated follow‐up data of the 2022 World Conference on Lung Cancer (WCLC) showed that for Stage II–IIIA patients with PD‐L1 ≥ 1%, the 5‐year overall survival (OS) in the treatment group was significantly improved (76.8% vs. 67.5%, HR = 0.71, 95% CI: 0.49–1.03). Compared with the BSC group, in the PD‐L1 high expression group (TPS ≥ 50%, excluding EGFR/ALK^+^), the 5‐year survival rate of the atezolizumab treatment group increased by 17.3% (84.8% vs. 67.5%, HR = 0.42, 95% CI: 0.23–0.78), significantly improved the prognosis of patients.

KEYNOTE‐091 [5] study data suggested that in adjuvant therapy, DFS improvement was observed in the whole population in the pembrolizumab group compared with placebo (HR = 0.76, 95% CI: 0.63–0.91, *p* = 0.0014), but in the population with PD‐L1 expression ≥50%, there was no statistical difference in DFS (HR = 0.82, *p* = 0.14). Subgroup analysis suggested that patients with squamous cell carcinoma, no adjuvant chemotherapy, and Stage IIIA had poorer benefit. Surprisingly, the benefit of the PD‐L1 high expression group was not as good as that of the whole population, and at the same time, the gene mutation‐positive group benefited more (small sample subgroup analysis), which is different from the results of Impower010. Prolonged follow‐up time can improve the benefits of PD‐L1 high‐expression population, and further follow‐up is therefore needed.

#### 2.1.2 Neoadjuvant immunotherapy

The CheckMate‐816 study [6] was the first randomized Phase III clinical trial to demonstrate that neoadjuvant immunotherapy combined with chemotherapy can significantly improve the rate of pathological complete remission (pCR) in patients with resectable NSCLC. The results of this Phase III clinical trial of nivolumab combined with platinum‐based chemotherapy versus platinum‐based chemotherapy alone showed that the pCR rate increased from 2.2% (95% CI: 0.6–5.6) to 24% (95% CI: 18.0–31.0), the event‐free survival was significantly improved, and the median EFS was 31.6 months versus 20.8 months (HR = 0.63, 95% CI: 0.43–0.91). The two survival curves were clearly separated after 2 years, which also means that patients receiving neoadjuvant immunochemotherapy may have long survival, but the OS data was not yet mature, and further follow‐up was needed. The latest data showed that patients who received neoadjuvant immunochemotherapy and achieved pCR had significantly longer EFS than those who did not achieve pCR (HR = 0.13, 95% CI: 0.05–0.37), but the EFS rate decreased significantly at 30 months. Therefore, whether it is necessary to extend the time of immunotherapy is a question that needs to be answered in follow‐up research.

At the recently held 2022 WCLC, the researchers announced the progression‐free survival (PFS) and OS results of the NADIM II study. The patients received the full regimen of neoadjuvant chemotherapy + surgery +  adjuvant immunotherapy, and the PFS was significantly prolonged. The median follow‐up time was 26.1 months. The 24‐month PFS rates of patients in the combination therapy group and chemotherapy alone group were 66.6% and 42.3%, respectively. The median PFS was not reached in the combination therapy group, while the median PFS in the chemotherapy alone group was 18.3 months (HR = 0.48, 95% CI: 0.25–0.91, *p* = 0.025) [6, 7]. The NADIM II study was also the first clinical study in which neoadjuvant chemotherapy showed OS benefits in patients with resectable IIIA‐B NSCLC, suggesting that perioperative immune neoadjuvant + adjuvant is expected to further improve the efficacy.

#### 2.1.3 Research on ctDNA as a potential biomarker

The 2021 European Society of Medical Oncology (ESMO) Congress published the results of ctDNA as a potential biomarker, showing that ctDNA positivity is associated with poor prognosis in DFS. Nonetheless, atezolizumab showed a DFS benefit in both ctDNA‐positive and ctDNA‐negative Stage II–IIIA patients. We also observed that the biomarker that affects the prognosis of patients was not ctDNA, but the expression level of PD‐L1. Since both ctDNA‐positive and negative patients benefit from immunotherapy, ctDNA cannot be used simply as a biomarker for adjuvant immunotherapy to screen the beneficiary population.

### 2.2 Immunotherapy for advanced NSCLC

In the field of advanced NSCLC, immunotherapy has made a historic breakthrough, rewriting the pattern of lung cancer treatment. Immunotherapy was first confirmed in the second line that it can improve the OS of patients with advanced NSCLC and became the second‐line standard treatment for patients without driver gene mutations [8, 9]. Then, the first‐line single‐drug immunotherapy for NSCLC patients with high expression of PD‐L1 also showed survival benefits, making the first‐line chemotherapy‐free strategy a reality [10]. To expand the beneficiary population of immunotherapy, immunotherapy combined with chemotherapy has achieved the coverage of the entire population of patients with advanced NSCLC with negative driver genes, and strategies such as combined immune and antivascular therapy and dual immunotherapy further enrich the means of immunotherapy.

#### 2.2.1 Combined immunotherapy and chemotherapy

The CameL study [11] was the first Phase III clinical study conducted on the Chinese population, enrolling patients with Stage IIIB or IV EGFR/ALK wild‐type nonsquamous NSCLC who had not received systemic treatment. The updated data showed that compared with chemotherapy alone, immunotherapy combined with chemotherapy significantly improved objective response rate (ORR 60.5% vs. 38.6%), and prolonged PFS (11.3 months vs. 8.3 months, HR = 0.60) and OS (27.9 vs. 20.5 months, HR = 0.73). The CameL‐sq study was camrelizumab combined with paclitaxel and carboplatin for the first‐line treatment of EGFR/ALK‐negative unresectable locally advanced or metastatic squamous NSCLC. The results showed that immunotherapy combined with chemotherapy was more effective than chemotherapy alone, significantly improved ORR (64.8% vs. 36.7%), and prolonged PFS (8.5 vs. 4.9 months). Compared with the chemotherapy group, the camrelizumab combined with chemotherapy group prolonged the median OS by nearly 1 year (27.4 vs. 15.5 months, HR = 0.57, 95% CI: 0.44–0.74, *p* < 0.0001) and significantly reduced the death risk by 43%, and the 2‐ and 3‐year survival rates were about 20% higher than those of the chemotherapy alone group (53.4% vs. 35.0%, 42.8% vs. 23.7%) [12].

The updated data of the KEYNOTE‐189 [13] study showed that the 5‐year OS rate of pembrolizumab combined with chemotherapy in patients with advanced adenocarcinoma patients with negative driver genes was 19.4%, which is very valuable data. The KEYNOTE‐407 [14] trial data showed that the 5‐year OS rate of patients with advanced squamous cell carcinoma was 18.4%, which is a major breakthrough in the treatment of advanced lung cancer. However, the data of the PD‐L1 < 1% subgroup is also worthy of attention. In the PD‐L1 ≥ 50% subgroup and the 1%–49% subgroup, the pembrolizumab regimen had shown obvious advantages, but there was no advantage in the PD‐L1 < 1% subgroup.

#### 2.2.2 Dual immunotherapy

The CheckMate227 [15] study published 5‐year follow‐up data, regardless of the patient's PD‐L1 expression level, compared with chemotherapy, nivolumab combined with low‐dose ipilimumab—dual antibody could bring durable and long‐term survival benefits. In patients with PD‐L1 ≥ 1%, the 5‐year OS rate of dual antibody versus chemotherapy was 24% versus 14%, HR = 0.77; while in patients with PD‐L1 < 1%, the efficacy of dual‐antibody therapy was more durable, 19% versus 7%, HR = 0.65, which is enough to reflect the advantages of dual immunotherapy and provide a choice for patients to go to chemotherapy‐free.

The results of the CheckMate 9LA [16] study showed that combined dual immunotherapy and chemotherapy compared with chemotherapy alone brought improved long‐term survival in PD‐L1‐negative patients. The 3‐year OS rate was 25% versus 15%, HR = 0.67. However, there was no difference in long‐term survival among PD‐L1‐positive patients, and the survival curves crossed. The above research results may mean that dual immunotherapy is more suitable for PD‐L1‐negative patients, and PD‐L1‐positive patients benefit more from immunotherapy combined with chemotherapy.

KEYNOTE 598 [17] compared the efficacy and safety of pembrolizumab combined with or without ipilimumab in the first‐line treatment of EGFR/ALK‐negative and PD‐L1 ≥ 50% Stage IV NSCLC. The results of the study showed that the dual‐antibody combination therapy failed to prolong the patient's survival time compared with the single drug (OS: 21.4 vs. 21.9 months; PFS: 8.2 vs. 8.4 months), but the toxicity was significantly increased (the incidence of TRAE above Grade 3: 35.1% vs. 19.6%). However, the main object of this study was the population with strong benefits of pembrolizumab, and the efficacy of PD‐1/PD‐L1 inhibitors combined with CTLA‐4 inhibitors in the population with low or no expression of PD‐L1 is still worthy of further exploration.

### 2.3 Novel immune combination therapy strategy

Immunotherapy is the standard mode of treatment for advanced NSCLC, and drugs such as PD‐1/PD‐L1 have been approved as first‐line monotherapy or in combination with chemotherapy for advanced NSCLC. However, in clinical studies, median PFS (mPFS) was less than 1 year, and a new treatment strategy is needed to further improve the outcome.

#### 2.3.1 Immunization combined with JAK inhibitors

The results of the Phase II clinical study announced at the 2022 WCLC Conference showed that pembrolizumab combined with Itacitinib (INCB039110, a JAK inhibitor) in the first‐line treatment of metastatic NSCLC with PD‐L1 ≥ 50% had a 12‐week ORR of 62%, and the mPFS was 23.4 months, achieving a meaningful and sustained ORR, which is expected to become a treatment trend in the future.

#### 2.3.2 Immunization combined with poly ADP‐ribose polymerase (PARP) inhibitors

Increased DNA damage by PARP inhibitors may alter tumor immunogenicity and sensitize tumors to PD‐1/PD‐L1 blockade, possibly triggering more durable PD‐1/PD‐L1 blockade than single‐drug antitumor response [18]. The 2022 WCLC conference announced the results of the ORION Phase II clinical study. Patients with EGFR/ALK wild‐type metastatic NSCLC and without disease progression (PD) in the initial treatment were randomly assigned to receive durvalumab combined with olaparib or durvalumab monotherapy as first‐line treatment. After a median follow‐up of 9.6 months, there was no significant difference in mPFS between the two groups (HR = 0.76, *p* = 0.074) [19].

#### 2.3.3 Immune combination antibody‐drug conjugate (ADC)

Datopotamab deruxtecan (DS‐1062a, ADC), a TROP‐2‐targeting ADC, showed encouraging efficacy as monotherapy in patients with relapsed/refractory advanced/metastatic NSCLC and manageable security features [20]. The Phase Ib study of TROPION‐Lung02 adopted the two‐drug or three‐drug combination therapy mode of Dato‐DXd combined with pembrolizumab ± chemotherapy. The results of the study showed that the security of the dual regimen was superior to that of the triple regimen. The chemotherapy‐related side effects of the triple regimen were significant, but there was no significant difference in the immune‐related side effects between the two regimens. The efficacy of immunocombination with ADC is encouraging. The overall remission rate of the first‐line treatment of the dual regimen was as high as 62%, and the ORR of the triple regimen was as high as 50%.

#### 2.3.4 Bispecific antibodies

With the improvement of drug development technology, the newly developed antibodies have been modified to recognize two or even three molecular targets, which are called tumor immune bi/trispecific antibodies. At present, the research and development of bispecific antibody drugs for tumor immunity emerges in an endless stream, mainly including T‐cell bridging bispecific antibodies (such as targeting CD3/EpCAM), bispecific antibodies based on PD‐1/PD‐L1‐based dual immune pathway regulation (such as targeting PD‐L1/CTLA‐4, PD‐L1/OX40, PD‐L1/CD47) and bispecific antibodies that block immune checkpoints and inhibitory molecules in the tumor microenvironment (such as targeting PD‐L1/TGF‐β) [21, 22].

AK112 (Ivonescimab) is a humanized anti‐PD‐1/VEGF bispecific antibody with an IgG1‐ScFv structure. It uses immune synergistic antiangiogenic strategies to simultaneously act on targets of PD‐1 and VEGF [23]. The results of Phase I clinical study showed that in the treatment‐naïve patients (*n* = 35) with PD‐L1 ≥ 1%, the ORR was 60%, and the disease control rate (DCR) was 97.1%. In the treatment‐naïve patients with PD‐L1 ≥ 50% (*n* = 13), the ORR was as high as 76.9%, and the DCR was 100%. With its unique “tumorimmunity + antiangiogenesis” dual‐targlkhfsaet synergistic antitumor mechanism, AK112 is expected to bring new hope to NSCLC patients who are drug‐resistant after PD‐1/PD‐L1 inhibitor therapy. At present, Phase III clinical trials have started, and the results are worth looking forward to.

## 3 IMMUNOTHERAPY FOR SMALL CELL LUNG CANCER (SCLC)

Due to the lack of corresponding driver genes in SCLC, platinum‐based doublet chemotherapy is the standard treatment option for extensive‐stage SCLC, making its treatment lackluster. With the advent of the era of immunotherapy and the deepening of the concept of precision therapy, the treatment of SCLC has also ushered in revolutionary changes.

### 3.1 Combination immunotherapy

The CASPIAN study was designed to evaluate patients with extensive‐stage SCLC, on the basis of standard platinum‐based doublet chemotherapy, further combined with an immune checkpoint inhibitor—durvalumab targeting PD‐L1, and further combined with an immune checkpoint inhibitor—tremelimumab targeting CTLA‐4, can bring corresponding survival benefits to patients [24]. The 2022 WCLC conference reported updated data: the benefit of durvalumab combined with chemotherapy compared with chemotherapy alone was still consistent, HR = 0.71, the median OS of the two groups was 12.9 and 10.5 months, respectively, and the 36‐month OS rates were 17.6% and 5.8%, respectively. Benefits or trends in benefits of the combination durvalumab were observed in each of the predicted subgroups. Compared with chemotherapy alone, dual immunotherapy combined with chemotherapy showed numerical benefits. The median OS of the two groups was 10.4 months (dual immunotherapy) and 10.5 months (chemotherapy alone), HR = 0.81, *p* = 0.02, and the 36‐month OS rates were 15.3% and 5.8%, respectively [25].

Phase III study KEYNOTE‐604 [26] suggested that, compared with placebo combined with EP regimen, pembrolizumab combined with EP regimen as first‐line treatment for patients with extensive‐stage SCLC can significantly improve the PFS of patients (HR = 0.75, 95% CI: 0.61–0.91, *p* = 0.0023), and the OS of patients also showed a trend of benefit but did not reach the significance threshold. The latest updated study data showed that for patients with previously untreated ES‐SCLC, compared with placebo + EP, pembrolizumab + EP still showed superior survival benefit improvement and manageability in long‐term follow‐up security. The 3‐year OS rate of patients in the pembrolizumab + EP group was 15.5%, compared with 5.9% in the control group, and patients who completed all treatment cycles could obtain better survival benefits. Based on the above data, the researchers believe that the application of the pembrolizumab combination strategy in patients with ES‐SCLC should continue to be explored.

### 3.2 PARP inhibitor combined with low‐dose temozolomide

Talazoparib (TALA) is a novel PARP inhibitor that exhibits cytotoxicity by inhibiting PARP proteins 1 and 2 and “trapping” PARP on DNA [27]. A Phase II open‐label, the single‐arm study aimed to investigate the efficacy and safety of TALA combined with temozolomide in patients with relapsed/refractory extensive‐stage SCLC who failed first‐line platinum‐based therapy. This study confirmed studies of the efficacy of PARP inhibitors and low‐dose temozolomide in SCLC, which exceeded the target response rate. Future Phase III studies will prove the advantages and disadvantages of this treatment mode and the currently approved treatment mode.

### 3.3 Bispecific T‐cell engagers show promising efficacy

Delta‐like ligand 3 (DLL3) is expressed in the vast majority of SCLC patients. Tarlatamab (AMG757) is a half‐life‐extended bispecific T‐cell engager (HLE BiTE®) that binds to DLL3 and CD3, leading to T‐cell‐mediated tumor lysis [28]. The DeLLphi‐300 study was the first human trial of tarlatamab, and the included patients were SCLC patients who had previously failed ≥1 platinum‐based treatment regimen. The results showed that in previously treated SCLC patients with a median of two prior treatments, the ORR of tarlatamab reached 23% (95% CI: 15.8%–33.7%), and the DCR was 55%. The median Duration of Response was 13 months, and the median OS was 13.2 months (95% CI: 8.8 to not reached). For previously treated SCLC patients, tarlatamab showed therapeutic promise and a manageable safety profile. Compared with other treatment methods for recurrent SCLC, PFS, and OS are comparable. Based on the results, a Phase II study of tarlatamab for the third‐line and above treatment of SCLC is underway.

## 4 SUMMARY

Immunotherapy has changed the landscape of lung cancer treatment. In NSCLC, single‐drug immunotherapy is suitable for patients with high PD‐L1 expression, and long‐term survival has been observed in such patients. In patients with high PD‐L1 expression, immunotherapy combined with chemotherapy can improve ORR, but not long‐term survival. For patients with low or negative PD‐L1 expression, combined immunotherapy and chemotherapy are recommended. The 5‐year survival rate can reach 18%–20%. Combined dual immunotherapy and chemotherapy are mainly suitable for PD‐L1‐negative patients, and dual immunotherapy can improve long‐term survival. In SCLC, ongoing and upcoming SCLC immunotherapy clinical trials include combinations of ICIs with novel targeted combinations such as PARP inhibitors, AKT1 inhibitors, and ATR inhibitors, and new immune‐based therapeutic strategies such as CART and BiTES, and so on, are expected to achieve good results. It is hoped that through the joint efforts of researchers, the 5‐year survival of advanced lung cancer will be further improved, achieving the goal of long‐term management of cancer as a chronic disease for more patients.

## AUTHOR CONTRIBUTIONS

**Qi Wang**: Writing—review and editing (equal). **Chunxia Su**: Resources (equal). **Caicun Zhou**: Investigation (lead).

## CONFLICT OF INTEREST STATEMENT

The authors declare no conflict of interest.

## ETHICS STATEMENT

Not applicable.

## INFORMED CONSENT

Not applicable.

## Data Availability

Not applicable.
